# “What matters to someone who matters to me”: using media campaigns with young people to prevent interpersonal violence and abuse

**DOI:** 10.1111/hex.12495

**Published:** 2016-11-03

**Authors:** Nicky Stanley, Jane Ellis, Nicola Farrelly, Sandra Hollinghurst, Sue Bailey, Soo Downe

**Affiliations:** ^1^ School of Social Work, Care and Community University of Central Lancashire Preston UK; ^2^ Faculty of Health Social Care and Education Anglia Ruskin University Cambridge UK; ^3^ School of Social and Community Medicine University of Bristol Bristol UK; ^4^ School of Health University of Central Lancashire Preston UK

**Keywords:** interpersonal violence and abuse, media campaigns, violence prevention, young people

## Abstract

**Background:**

While media campaigns are increasingly advocated as a strategy for preventing interpersonal violence and abuse, there is little evidence available regarding their effectiveness.

**Setting and design:**

Consultation with experts and young people was used as part of a UK scoping review to capture current thinking and practice on the use of media campaigns to address interpersonal violence and abuse among young people. Three focus groups and 16 interviews were undertaken with UK and international experts, and three focus groups were held with young people.

**Main results:**

Participants argued that, although campaigns initially needed to target whole populations of young people, subsequently, messages should be “granulated” for subgroups including young people already exposed to interpersonal violence and lesbian, gay, bisexual and transgender young people. It was suggested that boys, as the most likely perpetrators of interpersonal violence and abuse, should be the primary target for campaigns. Young people and experts emphasized that drama and narrative could be used to evoke an emotional response that assisted learning. Authenticity emerged as important for young people and could be achieved by delivering messages through familiar characters and relevant stories. Involving young people themselves in creating and delivering campaigns strengthened authenticity.

**Conclusions:**

Practice is developing rapidly, and robust research is required to identify the key conditions for effective campaigns in this field. The emotional impact of campaigns in this field appears to be as important as the transmission of learning.

## 1 Introduction

Interpersonal violence and abuse (IPVA) is a widespread, global phenomenon, and public health approaches are advocated as the optimum response.^1^, ^2^ Media campaigns are a key element in preventative public health approaches and have been utilized as part of the strategy to end violence against women in many parts of the globe.^1^, ^3^ Traditionally, campaigns that aimed to reduce IPVA have focused on raising awareness of violence against women in whole communities (see for example, Usdin et al.^4^) and/or encouraging victims to recognize their partner's behaviour as abusive and to seek help from appropriate services (see for example, Wray et al.^5^; Solomon et al.^6^). More recently, the focus has broadened to include perpetrators of IPVA and a number of campaigns in North America, Europe and Australia have targeted abusive men with the aim of encouraging them to seek help to change their behaviour.^7^, ^8^, ^9^

Bystander campaigns which seek to define interpersonal violence as a community problem and encourage both men and women to recognize and challenge IPVA among their peers^10^, ^11^ have emerged, and these campaigns have been delivered on university campuses in both North America and the UK. Social marketing techniques and theory which emphasize the value of formative research with the target audience, target segmentation and partnership with a range of stakeholders^12^, ^13^ have also become increasingly influential. This approach has informed a number of campaigns aimed at preventing IPVA.^9^, ^13^ Castelino et al.^14^ argue that adopting a social marketing approach to bystander interventions might prove a particularly effective means of changing social norms on masculinity and abusive behaviour.

However, although reviews of the evidence on the effectiveness of media campaigns in the field of public health have found that such campaigns have the potential to change health‐related attitudes and behaviour in large numbers of people,^15^, ^16^ evidence for the impact of these campaigns in addressing IPVA is limited. A review by the British Columbia Centre of Excellence for Women's Health^17^ undertaken to inform National Institute for Clinical Health and Care Excellence (NICE) Guidance on Domestic Violence and Abuse^18^ in the UK identified four evaluations of IPVA campaigns in the literature and found that evidence for effectiveness was mixed. The review concluded that while such campaigns have the potential to raise awareness of domestic violence and relevant services, their impact may be limited by implementation issues. As Noar^15^ notes, media campaigns need to be well designed and executed as well as appropriately targeted.

Some behaviours and audiences appear more amenable to change than others. Most of the campaigns discussed above address either whole populations or adult victims or adult perpetrators of IPVA. There is little in the published literature on media campaigns aimed at IPVA among adolescents, despite this group being a prime target for preventative IPVA interventions or “dating violence” programmes which are usually delivered in schools. Wakefield et al.'s review^16^ found that success was more likely when the behaviour targeted for change was one‐off or episodic rather than on‐going, and there are examples of campaigns that aimed to reduce drug or tobacco use among adolescents having the opposite effect to that intended.^19^, ^20^ This indicates that using media campaigns to address risky or abusive behaviour in young people may prove challenging. However, young people's high levels of engagement with a wide range of media have resulted in increasing interest in harnessing such campaigns to achieve change in this population.

A systematic review of preventative interventions in domestic abuse for children and young people,^21^ which was one element of the research reported here, found no studies in the peer‐reviewed literature addressing media campaigns for this audience. However, in the realm of practice, there are examples available of IPVA prevention campaigns designed for young people and some of these are reported via “grey” literature^22^, ^23^ or can be identified through online searches. The most notable of recent UK campaigns is the Home Office's *This is Abuse* campaign which achieved a high profile through the use of television and was delivered in several waves from 2010 onwards.

In the absence of an extensive *a priori* evidence base in this area, a multimethod study was undertaken to scope the current knowledge base on preventative interventions in IPVA for children and young people, where “preventative interventions” included both taught programmes and media campaigns. The study incorporated a systematic review of the international literature, a UK mapping survey and consultation with experts, policy makers and practitioners as well as with young people themselves. The results of the systematic review and the mapping survey, both of which focused on taught programmes, are reported elsewhere.^21^, ^24^ This paper reports on the consultations undertaken in relation to media campaigns that aim to prevent IPVA among children and young people. These findings of these consultations address the current gap in the literature and offer some key messages about current practice in, and stakeholder views about, media campaigns in this field as well as highlighting directions for future research. We draw on Rogers and Storey's^25^ work to define media campaigns as an organized set of communication activities which aim to produce specific outcomes or effects in a relatively large number of individuals.

## 2 Methods

As noted above, while the systematic review produced a considerable amount of published literature on programmes delivered in schools, only a small number of “grey” literature reports were found describing media campaigns. The absence of literature in this particular field where campaigns appeared to be developing rapidly indicated the importance of consultation to capture current practice and thinking. Consultation with relevant stakeholders was used to add rigour to the scoping review^26^ and offered a means of generating new data describing current policy and practice. The research was undertaken in partnership with two organizations that assisted in the task of convening and managing the consultation process. These were: Women's Aid which campaigns and provides services for women and children affected by domestic violence and the Personal, Social and Health Education (PSHE) Association which represents teachers delivering social and health preventative education.

Consultation groups were established with each meeting on three occasions over a period of 18 months. These included an expert group consisting of experts from the fields of IPVA campaigning, communications and health education; and a young people's group. The young people's group was convened from an existing group whose members had experience of being consulted on a range of social and health issues. Membership of the expert group was determined by drawing on the networks of both the research team and the partner organizations, with the aim of achieving a mix of policy, practitioner and researcher representation in each group as well as ensuring representation from all four countries of the UK.

The membership of both groups fluctuated between meetings. Eighteen young people aged 15–19 years attended the first meeting of this group, with seven or eight young people attending the two following meetings. While 10 members were present at the first meeting of the expert group, seven and subsequently six members attended the second and third meetings.

The consultation process was similarly structured for both groups, with topic guides used to structure all the groups. Participants were provided with feedback from the study that included progress reports and early findings, as well as being asked to discuss a series of key questions chosen to reflect the research questions which addressed the context in which campaigns were delivered, mechanisms of change, content and delivery, audiences and impact. A short clip from the UK Government's This is Abuse campaign was used to stimulate discussion in the young people's consultation group. As these were discussion groups and not focus groups, group interactions were not noted or recorded.

International perspectives were elicited through 16 interviews with international experts involved in the design, delivery or evaluation of preventative IPVA interventions for children and young people. These experts were selected by drawing on the knowledge and networks of the researchers, the study's two partner organisations and the expert consultation groups. While most of those interviewed were based in North America (5: USA, 3: Canada), Australia (4) or New Zealand (1), three UK experts who were unable to attend the expert consultation groups also took part in these interviews. Only one expert approached by the researchers declined to be interviewed and four did not respond to e‐mail requests. Most interviews were conducted by telephone; one interview was conducted face‐to‐face. A topic guide which allowed interviewees to reflect on essential themes including context, mechanisms of change and effectiveness of interventions was employed.

All consultation group members and interviewees were provided with appropriately formatted information about the study, and informed consent procedures were adopted which allowed for discussions and interviews to be audio‐recorded and transcribed. The young people's involvement was approved by the University of Central Lancashire's Ethics Committee.

Both interview and consultation group transcriptions were analysed thematically employing a framework whose structure derived from the main headings used for data extraction in the systematic literature review: context, theory, mechanism including delivery and content, audience and outcomes. Subthemes and new themes arising from the data were added as appropriate. Disagreements and variations in participants’ views within and between groups were identified. The software package NVivo (QSR International Pty Ltd., Doncaster, Australia) was used to assist with the sorting and storing of data.

## 3 Findings

### 3.1 Audiences

The diversity of audiences for campaigns aimed at children, and young people was emphasized across the consultations. Members of the expert group considered that campaigns should, in the first instance, target whole populations of children and young people but that subsequently, material should be “granulated” to reach subgroups. Campaigns that were multiplatform were useful in this respect but material could also be cascaded out to relevant target groups both in schools and through community organizations. Group members argued that, while raising awareness might be a sufficient outcome in some communities, for other groups, the task was to change abusive behaviour that was embedded in families and neighbourhoods; messages therefore needed to be distinguished for these different groups:

There is no one type of instigator… raising the empathy is enough for some young people… for others, particularly with the more disadvantaged, it's so ingrained, their lives are so much more complex, they haven't got that ability just to change their lives like that. (Expert Group 2)

One of the experts interviewed argued that the targeting of different subgroups required much fuller consideration:

…there's been very little attention so far to the ways in which we need to craft domestic abuse prevention in ways that address children or young people at a different risk of perpetration or being victimised… (Australia, Expert 3)

In the young people's group, there was disagreement as to whether campaigns should be targetted on particular groups. While the first speaker below argues for targeting interventions on vulnerable groups and perpetrators, the second suggests that distinguishing those children and young people who are most at risk would be difficult and potentially stigmatizing:

Participant 1: …those people who are, I'm going to use that word, vulnerable, from either being victims of it and the perpetrators… I think people with low, what is it, self‐confidence in themselves.

Participant 2: …it should be for everybody because I don't think you can pick out the vulnerable from the non‐vulnerable… You also isolate people if you take them out and say, well you're most vulnerable and you're the most likely to perpetrate. (Young People's Group 3)

There was uncertainty about whether campaigns should be gender specific. However, members of the expert group argued that preventative interventions were too often focused solely on female victims and that campaigns frequently failed to reach boys, who were the main perpetrators of abuse:

All the awareness campaigns that I've really had anything to do with have… been focused on raising awareness for victims, but not about raising awareness for young people who might be using [that] behaviour… (Expert Group 1)

Likewise, experts interviewed identified a shift from an earlier focus on victimhood to addressing perpetrators. Rather than equipping girls to be more assertive, the aim was now to change boys’ behaviour:

…really it's totally up to whoever might victimise them to change their behaviour…Primarily, you want to target potential perpetrators… (USA, Expert 1)

One interviewee involved in developing campaigns argued that the focus had switched to providing boys with knowledge about what girls wanted in a relationship as what motivated boys was the possibility of being successful in attracting a girlfriend.

Furthermore, both individual experts and expert group members noted that preventative campaigns “tend to be very heterosexual focused” and it was argued that that interventions should recognise and speak to Lesbian, Gay, Bisexual and Transgender (LGBT) young people and promote talking “about the different relationships that we can have” (Expert Group 1).

While it was felt to be important to reach a range of ethnic groups, expert group members noted that it could be a struggle to address diversity in a short media advertisement lasting less than a minute without risking associating domestic abuse with a particular ethnic group. It was felt to be easier to reach minority or subgroups through developing associated materials that targeted them or could be used in particular settings such as youth groups or young offenders’ institutions or groups.

### 3.2 Using drama and narrative

Members of the Young People's Group emphasized the value of incorporating drama and/or narrative into campaigns. The use of drama allowed for emotional identification with the characters which “made it real” for a young audience. The *This Is Abuse* campaign which used young characters from a popular television soap series was cited as an example that promoted such identification:

…because of our like age group, we could relate to it a bit more, it seems more real. (Young People's Group 2)

Drama, narrative and real‐life accounts could invest a campaign message with immediacy, relevance and authenticity:

It's like in front of you and then you realise, actually, it doesn't happen miles away, you know, it happens here. And it's so close to home and it happens to people that you might know and, you know, it can easily happen to anyone. And so I think drama kind of conveys that a bit more. (Young People's Group 3)

Members of the expert group also identified a strong narrative as a central feature of a successful campaign. In common with effective commercial advertising aimed at teenagers, such narratives needed to be solution focused and worked by:

…telling the story, saying, you know, this is a problem you might encounter, this is how you solve it. (Expert Group 2)

Group members agreed that a story could achieve its effect by evoking emotional engagement:

it works because of the emotional engagement, it's not the fact that it's a story, it's the fact that it engages in emotion … Whatever engages people with the heart as well as the head is probably going to be effective. (Expert Group 1)

In this sense, emotion was seen as an aid to learning: impact was achieved through the visceral charge that accompanied a message.

Expert group members agreed with the young people's group in identifying authenticity as a key component of an effective campaign. Delivering messages through characters who appeared regularly in television soaps, who were familiar to young people and who young people cared about could ensure that they were perceived as authentic:

you already know the character, if it's someone that's in their house every day that they recognise…that has… the integrity of a celebrity that [you] genuinely believe, believe more, the character that you sort of know and love. (Expert Group 1)

Similarly, one member of the young people's group summarized the use of television soap stars to deliver effective campaign messages as it “*matters to someone who matters to me”* (Young People's Group 1).

Celebrities, such as sports people and musicians, including online rap artists, had been used by campaigning organizations to spearhead and disseminate campaign messages. However, expert group members emphasized the need for them to be perceived as genuine. There were risks, which were reiterated by the young people's group, that celebrities could be perceived as pursuing their own agendas or that they could deliver conflicting messages. Young survivors of domestic abuse who were known and recognizable to young people could provide powerful role models and had credence. Male survivors were considered particularly valuable in that they were able to address young men's perspectives.

### 3.3 Involving young people in campaign creation and delivery

There was considerable enthusiasm expressed across the consultations for involving young people themselves in developing and delivering campaigns. One of the experts who was involved in developing online campaign material argued that young people's use of the Internet to access preventative material could be construed as active engagement in a campaign:

…because young people are already using the internet…that is not a passive kind of consumption…you're actually utilizing young people in spreading messaging and kind of participating in really absorbing the prevention part of it. (USA Expert 5)

Active participation in campaigns was considered to enhance learning—”people learn better by doing” (Young people's consultation group 1)—and one member of the young people's group who had been part of a “flashmob” dance performed globally on the International Day for the Elimination of Violence against Women emphasized both the participative benefits and the public recognition as contributing towards a momentum towards change:

…when you do something and it's on more of a national level… you like feel like you're a part of something, like obviously the dancing, there are people doing it all over the world, so you felt like it's a worldwide movement. (Young People's Group 1)

Members of the expert group stressed that the language and content of campaign material was more likely to be perceived as relevant by young people if they had contributed to its development and argued that delivering preventative messages to young people through their peers replicated young people's natural tendency to look to their friends for support. Involving young people in the production of the campaign was perceived as a means of embedding and extending the reach of campaign messages:

There's some really good examples of co‐design work that's youth led and that, that has really good impact, because you effectively create evangelists, you create disciples for your issue… (Expert Group 2)

Co‐production of campaigns could extend to ceding control to young people, and an example of this was provided by a campaign funded by the UK's Department of Health that used young people who were already providing regular advice to subscribers through online video blogs to produce a series of online videos addressing difficulties in intimate relationships. It was noted that young people's ownership of new technology made a shift towards such co‐creation initiatives more likely:

Technologies democratise communications to the extent that young people… instead of just consuming content themselves, they are now architects of that, they are making it, and we have to accept reality that we can't retain all control, we have to allow conversations to happen… (Expert Group 2)

However, some experts emphasized the need for a balance between “soliciting those ideas but also giving them the guidance about where they're going” (USA, Expert 5) when working collaboratively with young people to create campaigns.

It was agreed that organizations needed to evoke the trust of young people who were to be involved in developing campaigns. The importance of establishing trust between young people and the information source was identified in a number of meetings of the expert group, and it was noted that the format in which a message was delivered could impact on that trust. For example, young people were often sceptical about advertising, which could affect how a campaign was viewed:

…we can't be seen to be advertising… That's the problem. We're using, a lot of the time, the same mechanisms, but if we're seen as advertising you immediately lose trust and genuineness. (Expert Group 3)

### 3.4 Evaluating campaigns

Members of the expert group agreed that currently, there was little robust evidence on what made campaigns effective. Underfunding and short‐term funding were considered to have contributed to a lack of good quality evaluations and those designing new campaigns either drew on the experience of those who had worked on previous campaigns or tended to “reinvent the wheel” by starting from scratch.

However, the methodological challenges of measuring the impact of media campaigns in this field were also obstacles to building an evidence base. The problem of delivering a campaign in a media environment rife with confounding influences or “noise” was identified as a key issue:

You can't necessarily say with 100% confidence all the time that it's your campaign that is moving the needle either way, right? Because there's a lot of other noises out there. (USA Expert 5)

The expert group identified a range of outcomes that might be adopted to measure change. An increased readiness to discuss IPVA openly at a social level was identified as a key outcome, and this was linked to the group's view that media campaigns should be aimed at facilitating discussion of topics that were previously taboo so they could be discussed openly “around the water cooler” (Expert Group 3). Increased recognition of abusive behaviour in the individual should provoke help‐seeking or service use and these could be used as indicators of change. Among perpetrators, increased empathy for others was a desirable outcome which should be measured.

However, those consulted agreed that campaigns should also aim to reduce abusive and violent behaviour as well as increasing social and individual awareness. Members of the expert group suggested that campaign evaluations should measure subsequent disclosures and reports of IDVA which would be expected to rise initially as individual awareness increased, with a reduction in incidence in the longer term. Some of the experts interviewed, particularly those from Australia, New Zealand and the UK, were interested in finding new approaches to measuring behavioural change that would include indicators of ability to negotiate sexual consent.

## 4 Discussion

The findings from these consultations indicate that practice in relation to using media campaigns in IPVA prevention for young people is developing rapidly, boosted in part by the rapid growth of young people's access to a range of media. Those working in this field and young people themselves have strong views, derived from an increasing body of experience, about what makes for a successful campaign. There is however limited research evidence concerning the effectiveness of media campaigns in changing young people's behaviour in respect of IPVA.

As those participating in this study noted, the question of audience segmentation appears key for these campaigns. While most young people hold positive attitudes condemning IPVA^27^ that can be harnessed by whole population campaigns, and which bystander campaigns make explicit use of, those who have grown up in communities where IPVA is widespread and embedded may need messages that target their beliefs and experiences more precisely. Gadd et al.^28^ studied the responses of young men who had personal histories of high levels of violence and abuse to a viewing of a film produced as part of the Home Office's *This is Abuse* campaign. When encouraged to explore their responses in depth, the research participants revealed conflicting views and abusive values and attitudes were found to coexist alongside the condemnation of violence that the campaign was designed to evoke. While the participants’ initial reaction indicated that they had recognized that the socially acceptable response was condemnatory, eliciting their fuller responses suggested that their behaviour was unlikely to change significantly.

Participants in the study reported here noted the lack of materials for LGBT young people, and there is evidence that the prevalence of IPVA in LGBT communities is similar to that in heterosexual groups.^29^ The needs of young LGBT people may be particularly urgent as emotional abuse of partners may be compounded by the threat of unwanted “outing” which may be especially harmful for young people struggling to define their sexuality. Moreover, disclosure rates are lower, and consequently, sources of support may be harder to access for this group.^30^

The consultations also raised the question of whether campaigns should be gender specific. There is increasing interest in targeting campaigns on boys since, as they are more likely to be perpetrators of the most serious forms of abuse and violence that are sustained and inflict harm,^31^ it is generally boys’ behaviour that requires change. Such campaigns will need to deliver a message that is positive rather than blaming or alienating if they are to be acceptable to a young male audience.^28^, ^32^ Using narrative or drama to deliver such messages in an authentic manner that feels “real” to young people may be effective, but the trust of young people can be difficult to elicit and retain, and there are risks entailed in using celebrities or in imitating the formulas of commercial advertising.

The centrality of authenticity for an adolescent audience strengthens the argument for involving young people themselves in the creation of campaigns. It is however, also important to bring understanding of the mechanisms of change to the development and evaluation of campaigns. If campaigns work by shifting social norms,^33^ peer involvement and attitudinal change are important to achieve. In this respect, social marketing approaches which emphasize the value of campaigns engaging with the target audience from the outset are particularly relevant. If campaigns are seeking to elicit identification with and empathy for the victim, emotional engagement with a resonant narrative may be what makes for behavioural change. There is as yet insufficient understanding of *how* media campaigns effect change in violent and abusive behaviour. In addition to measuring effectiveness, research needs to elucidate the process of change and explore the possibility that more targeted and attuned messages are required for those groups for whom abusive behaviour is embedded and habitual.

## 5 Conclusion

Enthusiasm for media campaigns as a vehicle for ending violence to women needs to be underpinned by robust research addressing what works, for whom, and in what circumstances. For respondents in this study, notions of authenticity and the need for tailored messaging were captured in the phrase “*what matters to someone who matters to me*.” Cynical co‐option of celebrities and popular tropes into campaigns was reported to be counterproductive as authenticity was a key to young people's engagement in campaigns. There was widespread support for the active engagement of young people themselves in the process of creating relevant campaigns, and this was envisaged as extending beyond social marketing's conception of research or consultation with the target audience to including young people as co‐producers of campaigns. Affective messages were reported to be at least as important as the transmission of knowledge.
